# Enantiomer discrimination in β-phenylalanine degradation by a newly isolated *Paraburkholderia* strain BS115 and type strain PsJN

**DOI:** 10.1186/s13568-018-0676-2

**Published:** 2018-09-21

**Authors:** Oliver Buß, Sarah-Marie Dold, Pascal Obermeier, Dennis Litty, Delphine Muller, Jens Grüninger, Jens Rudat

**Affiliations:** Section II: Technical Biology, Institute of Process Engineering in Life Sciences, Karlsruhe Institute of Technology (KIT), Engler-Bunte-Ring 3, 76131 Karlsruhe, Germany

**Keywords:** β-Phenylalanine, ω-Transaminase, Kinetic resolution, Chiral resolution, *Paraburkholderia* sp., Fermentation, Acetophenone degradation

## Abstract

Despite their key role in numerous natural compounds, β-amino acids have rarely been studied as substrates for microbial degradation. Fermentation of the newly isolated *Paraburkholderia* strain BS115 and the type strain *P. phytofirmans* PsJN with β-phenylalanine (β-PA) as sole nitrogen source revealed (*S*)-selective transamination of β-PA to the corresponding β-keto acid by both strains, accompanied by substantial formation of acetophenone (AP) from spontaneous decarboxylation of the emerging β-keto acid. While the PsJN culture became stationary after entire (*S*)-β-PA consumption, BS115 showed further growth at a considerably slower rate, consuming (*R*)-β-PA without generation of AP which points to a different degradation mechanism for this enantiomer. This is the first report on degradation of both enantiomers of any β-amino acid by one single bacterial strain.

**Electronic supplementary material:**

The online version of this article (10.1186/s13568-018-0676-2) contains supplementary material, which is available to authorized users.

## Introduction

The catabolism of proteinogenic l-alpha-amino acids (l-α-AA) is in-depth understood and part of undergraduates’ biochemical education (Voet and Voet 2011). In contrast, bacteria have long been known to be a rich source of d-α-aa which are therefore abundant in soil and fermented food (Brückner and Hausch 1989). The bacterial synthesis mechanisms of d-α-AA and their incorporation in non-ribosomal peptides are now well-understood (reviewed by Radkov and Moe 2014). However, in some cases the enzymes for these reactions are still unknown. Furthermore, not only l-α- and d-α-AA are present in nature and reactions towards amino acids with amino groups in other positions than α-position are possible, leading to β-, γ-, ε- and other constitutional isomers of amino acids. ε-amino acids have e.g. a special significance in the biosynthesis pathway of β-lactams in actinomycetes and are converted by lysine ε-aminotransferases (Duan et al. 2016).

By contrast, only minor insight has been gained into the degradation of β-amino acids (β-AA). With the exception of β-alanine and β-aminoisobutyric acid which constitute key intermediates in several metabolic pathways, β-AA are not as abundant in nature as their α-configured counterparts. β-alanine can be metabolized by mammalia (Nutzenadel and Scriver 1976) and is involved in pantothenate metabolism (Spitzer et al. 1988), finally becoming a component of the key metabolite coenzyme A (CoA). β-alanine is also linked with histidine by carnosine synthase to the dipeptide carnosine which is abundant in skeletal muscles as well as in the central nervous system of most vertebrates (Drozak et al. 2010). Using the KEGG-pathway tool for analyzing the degradation of β-alanine, it can be seen that a transaminase reaction is forming malonate-semialdehyde which is further converted to malonate and finally enters the fatty acid biosynthesis as malonyl-CoA (Kanehisa et al. 2016).

However, β-AA have been recognized as essential parts in a variety of biologically active compounds. Beside the incorporation of β-AA in non-ribosomal peptides and their polyketide hybrids by several bacteria, it has also been shown that plants produce β-AA containing natural compounds, with the terpenoid-β-AA hybrid Paclitaxel as the most prominent example (better known as the anticancer therapeutic Taxol) (Wani et al. 1971). In the last two decades, β-AA also raised interest as building blocks for β-peptides, which are able to form protease-resistant, stable and predictable secondary structures and thus promise applications as peptidomimetics (Seebach and Gardiner 2008; Steer et al. 2002). Furthermore, cyclized and substituted β-AA like β-lactams constitute the most common antibiotics and also show additional encouraging pharmacological properties; moreover, some free β-AA show pharmacological effects such as antifungal agent (Ziegelbauer et al. 1998; Magriotis 2001; Juaristi and Soloshonok 2005). In addition, aromatic β-amino acids might be useful for the creation of artificial flavors like tobacco aroma (Tao et al. 2017). The synthesis of β-AA and their incorporation into natural compounds has been extensively reviewed by Kudo et al. (2014). Microorganisms appear to be routinely affected with β-AA. Thus a deeper understanding of degradation mechanisms promises (A) an insight into defense mechanisms of microorganisms affected with these natural compounds (B) environmental aspects referring to the persistence of β-AA in soil and water (C) pharmacokinetics of these natural compounds when used as a drug, e.g. cytostatics containing aromatic β-AA.

The production of chiral β-AA poses a major challenge in regard to synthetic efficiency and in regard to the used strategy. The solutions include enantioselective synthesis by complex organometallic catalysis, but also by a few enzymatic asymmetric synthesis methods, which must ultimately be more efficient than pure kinetic resolution processes (Juaristi and Soloshonok 2005). Therefore mainly deracemization synthesis strategies were presented on industrial scale as shown by Evonik-Degussa (Grayson et al. 2011). In this strategy, a racemic ester is first produced which is then cleaved in a further reaction by an enantioselective lipase to obtain only one enantiomer as amino acid. The disadvantage is that without racemization (and subsequent dynamic kinetic resolution) the remaining ester enantiomer is usually produced as waste. However, the advantage is that the starting racemate can often be produced in large quantities with little effort, so that chiral resolution turns out to be economically feasible anyway.

Therefore, chiral β-AA products can be also obtained from enantioselective microbial degradation processes. In case of a deamination to β-keto acids, the reaction equilibrium is shifted by spontaneous decarboxylation of the products which will be a major subject of this study. For β-phenylalanine (β-PA) as an example for the chiral β-AA synthesis, a chemical synthesis from benzaldehyde with malonic acid and ammonium acetate to *rac*-β-PA would be a starting point for deracemization, as already shown by Grayson et al. (2011). Afterwards, the product could be deracemized by kinetic resolution using an enantioselective transaminase. A fermentation processes might be an alternative to the use of isolated enzymes (Yun et al. 2004; Crismaru et al. 2013; You et al. 2013). In a previous study we performed enrichment cultures from soil samples using aromatic β-PA as sole nitrogen source (Brucher et al. 2010b). Transamination was found to be the predominant initial degradation step in all isolated bacteria: biotransformation with cell-free extracts and fractions thereof was only observed when a suitable amino acceptor was additionally applied (preferably α-ketoglutarate or pyruvate) as well as the well-known transaminase cofactor pyridoxal phosphate (PLP). The fermentative degradation of *rac*-β-PA by living cells has only once been documented by Mano et al. in (2006). The (*S*)-selective ω-TA involved in this degradation has been characterized in detail by Hibi et al. (2012). However, the further metabolization of the desaminated (*S*)-β-PA (and thereby created β-keto acid, β-KA) in the degrading microorganism remains as uncertain as the fate of the (*R*)-β-AA. Here we report the degradation of racemic β-PA by the newly isolated *Paraburkholderia* strain BS115 and the *Paraburkholderia* type strain *P. phytofirmans* PsJN. We document the degradation of both enantiomers by the first strain and only one enantiomer by the latter, following product generation and gaining a first insight into the responsible enzymatic mechanisms.

## Materials and methods

### Chemicals

All amino acids and chemicals were purchased from Sigma Aldrich (St. Louis, US) and Carl Roth (Karlsruhe, Germany) in analytical grade. All enantiopure amino acids were purchased from PepTech Corporation (Bedford, US).

### Bacterial strains

*Paraburkholderia* BS115 was isolated by enrichment culture from garden soil spiked with soy peptone due to its high content of aromatic amino acids (Brucher et al. 2010b). The strain was identified by the culture collection DSMZ (Deutsche Stammsammlung für Mikroorganismen und Zellkulturen GmbH) as *P. phytofirmans*. Cultures of the strain were deposited at the DSMZ designated as DSM 103130 *P. phytofirmans* (BS115). *P. phytofirmans* PsJN was also delivered by DSMZ (DSM 17436).

### Fermentation process

The sterile minimal medium M1 (pH 7) contained 100 mM d-glucose and 10 mM of *rac*-β-PA, 5.8 mM KH_2_PO_4_, 4.1 mM NaHPO_4_·2 H_2_O, 1 mM MgSO_4_·7 H_2_O, 0.5 mM CaCl_2_·2 H_2_O and 0.01 mM FeCl_2_·4 H_2_O. Additionally the vitamins pyridoxal-5-phosphate (PLP) and cobalamin were added to the mixture in concentrations of 1 µM and 1 nM, respectively. The vitamins, β-phenylalanine and FeCl_2_ were sterile filtrated separately while all other compounds were autoclaved. The sterile compounds were then mixed. The fermentation process was optimized for maximal growth rate in a small scale bioreactor system without pH regulation.

The fermentation of PsJN and BS115 was performed in a benchtop reactor (vessel volume 2.5 L; Minifors, Infors-HT, Switzerland) with a working volume of 1 L in minimal medium M1. The system was equipped with a pH probe (Mettler-Toledo, USA) and a Pt-100 temperature probe. The temperature was set to 30 °C and the stirrer speed was adjusted to 120 rpm at the beginning. For mixing and disruption of gas bubbles a standard Rushton stirrer (diameter 46 mm) was used. The pH-value and pO_2_ content were not controlled, but monitored during the fermentation. The aeration rate was set to 1 L/min. The experiment was conducted in a triplicate. The precultures were grown in a 100 mL shake flask in the minimal medium at the same temperature and inoculated from a glycerol stock. The precultures were cultivated in a rotary shaker (Infors-HT, Switzerland) at 120 rpm. The bioreactors were inoculated with the precultures to an optical density at OD_600nm_ of at least 0.1.

### Dry cell mass measurement

10 mL of the cultures were centrifuged at 6000*g* for at least 10 min at 4 °C. The pellets were washed with _HP_H_2_O (high-purity water, type I ISO 3696). After this, the cell pellets were dried overnight at 60 °C in a drying oven.

### Glucose assay

The concentration of d-glucose was measured by an enzymatic glucose-assay from R-Biopharm AG (Darmstadt, Germany). According to the manufacturer’s assay protocol volumes were down-scaled to 96-well microtiter plates (scale 1:20). 5 µL of the sample was mixed with 100 µL _HP_H_2_O and 50 µL of solution 1. 10 µL of 1–10 diluted suspension 2 was pipetted to the mixture. The absorption at 340 nm of the incomplete mixture was measured in an epoch plate reader system before and after suspension 2 was added to the mixture. Also after 5 and 10 min the absorption was measured. The measurement was calibrated by a d-glucose dilution series from 0.01 to 1 g/L.

### Enzyme activity assay

Both strains were cultivated for at least 3 days at 120 rpm and 30 °C in 100 mL minimal medium in shake flasks, the final OD_600_ was 3.4 for BS115 and 3.1 for PsJN. The cells were harvested by centrifugation at 8000 rpm in Beckman Coulter (Brea, USA) centrifuge (JA-10 rotor) and lysed by incubation with 1 mg/mL lysozyme (Fluka) for 15 min at room temperature. Pellets were resuspended in 5 mL 40 mM ice cold sodium phosphate buffer (pH 7.2) and sonicated for 5 min with 30 s pulsations at 50% amplitude. The cell lysate was clarified by centrifugation at 50,000*g* for 30 min at 4 °C (Beckman Coulter). The cell free supernatants were used for testing the enzyme activity of both strains: 100 µL of lysate were added to 100 µL of reaction solution. The consumption rate was normalized by the protein concentration (standard Bradford test) of both lysates (Bradford 1976). The activity test solution contained 15 mM of *rac*-β-PA and 15 mM α-ketoglutaric acid (amino acceptor). Additionally, 0.1 mM of the cofactor PLP was added to the solution. The solution was buffered by 40 mM sodium phosphate and adjusted at 7.2 with HCl. The reaction temperature was set to 30 °C and samples were taken at various points. The enzymatic activity was defined as 1 U = 1 µmol of converted β-PA per min. The samples were diluted with preheated sodium phosphate buffer (40 mM, 1 mM l-leucin, pH 7.2) and incubated at 99 °C for 5 min to stop the reaction. l-leucin was used as internal standard for HPLC analysis.

### Quantification of β-PA by HPLC

The samples of the fermentation process were centrifuged for at least 5 min at 13,000 rpm in a bench top centrifuge (Eppendorf, Germany) and used for quantification of β-PA and AP. The supernatant was used for reversed phase HPLC analysis (Agilent 1200 Serie) by automated precolumn derivatization with *ortho*-phtaldialdehyde and *N*-isobutyryl-l-cysteine to obtain diasteromers of β-PA for chiral separation (Brucher et al. 2010a). Therefore, a C18 column (150 × 4.6 mm HyperClone 5 µm. Phenomenex Inc., Aschaffenburg, Germany) was used. The mobile phase was a mixture of 55% methanol and 45% of 40 mM sodium phosphate buffer (pH 6.5). The flow rate was set to 1 mL/min and the column temperature to 40 °C. The diastereomers were monitored by UV-light absorption at 337 nm. The injection volume of the samples for the derivatization mixture was set to 0.5 µL. The total injection volume of the derivatization mixture was 10 µL. Calibration was performed using *rac*-β-PA (0–15 mM). To determine (*R*)- and (*S*)-enantiomers we used optically pure β-PA standards from Peptech (Burlington, USA).

### Quantification of acetophenone (AP) by HPLC

No precolumn derivatization was performed to determine acetophenone in samples. Samples were centrifuged for at least 5 min at 13,000 rpm in a bench top centrifuge. The injection volume of AP was 5 µL. AP was monitored at 245 nm using a C18 column (Kinetex 5 µm EVO C18 150 × 4.6 mm). The mobile phase was not changed compared to β-PA analysis.

### Quantification of d-glucose by HPLC

For quantification of d-glucose in PsJN samples, HPLC was used. This was necessary, because samples showed an interaction with the enzymatic assay. Glucose concentration was measured using an HPLC-system with RI-detector system (Agilent 1100 series). The protocol was adapted from Buchholz et al. (2014). Briefly, samples [S] were precipitated by 4 M NH_3_ in combination with 1.2 M MgSO_4_ in volumetric ratio of 0.87 [S]: 0.039 (NH_3_): 0.087 (MgSO_4_), incubated for a few minutes at RT and centrifuged at 17,000*g* in benchtop centrifuge. The supernatant was mixed 1:1 (v/v) with 0.1 M H_2_SO_4_ and was again incubated at RT for at least 20 min and centrifuged at 17,000*g* for 15 min. 10 µL of the supernatant were injected on a Rezex ROA-organic acid H^+^ column (300 × 7.8 mm, Phenomenex). The mobile phase consisted of 5 mM H_2_SO_4_ solution with a flow rate of 0.4 mL/min. The temperature was set to 50 °C for the column and 32 °C for RI-detector. For calculation of the d-glucose concentration, a calibration curve was measured from 0.01 to 0.5 g/L.

### Data analysis

The maximal growth rate µ and substrate-consumption were fitted using a sigmoidal equation with three variable parameters to the optical density/dry-cell mass of the cell cultures and to the concentration of β-PA during the fermentation with data analysis software Sigma Plot (San Jose, USA) adapted to Dörsam et al. (2017). The parameters a and b were defined as variables for the sigmoidal equation fit using Sigma Plot. x_0_ can be interpreted as half maximum of biomass production or as the half amount of the consumed β-PA and is the inflection point of the function. The variables are x and f(x). The cultivation time was defined as x in hours. The corresponding f(x) was defined as optical density, dry cell mass or the concentration of β-PA (mM).

$$f\left( x \right) = \frac{a}{{1 + e^{{ - \frac{{x - x_{o} }}{b}}} }}$$

After fitting of the function against the measured values, the maximal rates were determined by calculating the slope using numeric differentiation with Excel (Microsoft, USA).

## Results

Both strains BS115 and PsJN were able to grow in minimal medium containing β-PA as sole nitrogen source in 1 L scale experiments. The fermentation process was started by inoculation of β-PA induced precultures.

### Fermentation of *Paraburkholderia*

**Fig. 1:**
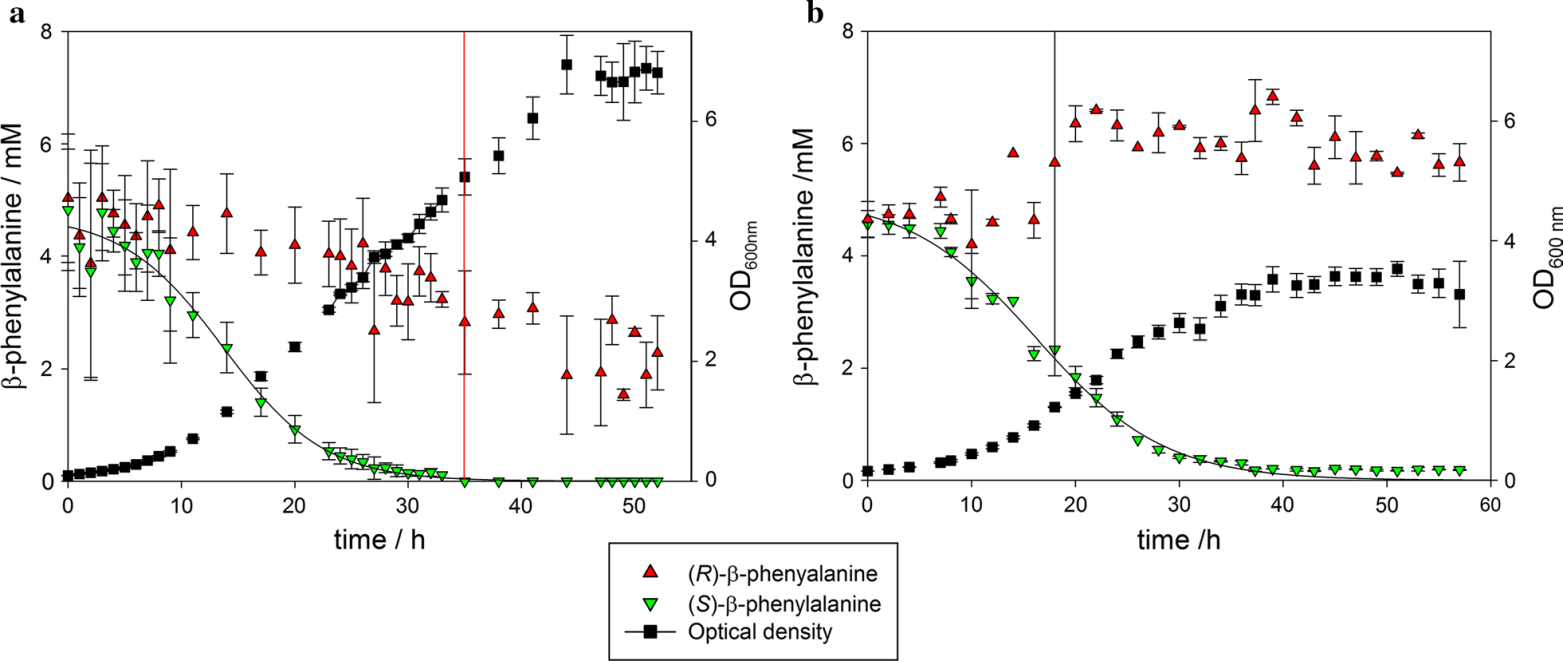
Degradation of *rac*-β-phenylalanine by *Paraburkholderia* sp. in a 2.5 L bioreactor. The fermentation was conducted in triplicate at 30 °C in 1 L minimal medium M1 with 10 mM of *rac*-β-PA. **a** BS115, **b** PsJN. The red line indicates (*S*)-β-PA < 1%

The main fermentation process of BS115 is illustrated in Fig. 1a. The lag phase of the culture was observed between 0 and 12 h. After this incubation period the culture was growing with maximal growth rate (12–21 h) until (*S*)-β-PA was almost completely consumed. After the preferred enantiomer was depleted, growth continued at a considerably slower rate. The stationary phase was reached 48 h after inoculation. The total consumed amount of (*R*)-β-PA was 2.5 mmol after 50 h of fermentation. In contrast the AP concentration increased with progressing fermentation and peaked at 4.5 mmol, when (*S*)-β-PA was almost fully consumed thereby nearly matching the consumed concentration of (*S*)-PA. Thereafter the AP concentration substantially decreased until the stationary growth phase was reached (Additional file 1: Figure S1).

Growth resulted in a maximal optical density of 7.0, resembling a dry cell mass (DCM) concentration of 1.8 g/L at the end of the fermentation. The oxygen concentration before inoculation was set to 100% and dropped during the process to a minimum of 80%. While centrifuging fermentation samples for analytical purposes, the formation of a slime layer was observed when (*S*)-β-PA was fully consumed, which reproducibly happened at each fermentation. The extracellular capsule built by BS115 was visualized by negative contrasting with Chinese ink (Additional file 1: Figure S2).

After 24 h the pH-level dropped from pH 7.0 to pH 6.0, then remained constant for 20 h, but decreased in the last 10 h to a final pH of 3.5 (Additional file 1: Figure S4). The remaining concentration of d-glucose inside the reactor was 27 ± 1.6 mM and total amount of consumed d-glucose was 73 mM. The ratio between consumed carbon to consumed nitrogen (C:N) was calculated as 14.6 mM per 1 mM of (*S*)-β-PA.

We also investigated the fermentation process of PsJN to characterize β-PA degradation. Furthermore PsJN was used as control strain, to exclude that the degradation of (*R*)-β-PA is only an unspecific effect during the fermentation process e.g. by adsorption of the amino acid to bacterial cell envelopes or due to other reasons.

The fermentation parameters were maintained according to the BS115 fermentation. The degradation of the (*S*)-enantiomer was almost complete after 38 h, which is in total 5 mM of β-PA (Fig. 1). During the whole fermentation no degradation of (*R*)-β-PA was observed. The AP concentration increased during the process to a maximum of 1.6 mM after 35 h (Additional file 1: Figure S1). After 50 h a slow decrease of the AP concentration in the medium to 0.8 mM was determined. In addition, we also observed only a slight capsulation of the microorganism with increasing fermentation time, which was not observed in shake flask experiments. The total amount of consumed (*S*)-β-PA was three times higher than the maximal concentration of AP in the medium. The OD_600nm_ reached a maximum of 3.3 which is half the OD_600_ reached in the BS115 fermentation. The oxygen concentration decreased to a minimum of 70%. In contrast to BS115, the pH-value dropped from neutral (pH 7) to pH 5, instead of pH 3.5. The d-glucose concentration could not be determined by glucose assay, caused by an inhibition or unknown interaction of the enzymatic assay with the supernatant of the fermentation medium. Therefore, we determined the concentration of d-glucose by HPLC. The remaining concentration of glucose inside the fermentation medium was 21.9 mM ± 2.5 mM after 50 h, which is 5 mM less than for BS115. The molar ratio of consumed carbon to nitrogen source was 15.6 per 1 mM (*S*)-β-PA. The DCM peaked after 50 h of cultivation at the concentration of 0.9 g/L, which is only half as much as for BS115.

Within the 2-day fermentation of BS115, the DCM value of 2 g/L is significantly higher than with PsJN, but the Y_x/s_ value of 0.15 is rather low in relation to the amount of biomass produced in relation to the amount of depleted glucose. Surprisingly a consumption of the (*R*)-enantiomer of β-PA was shown for BS115 but the growth was limited although neither (*R*)-β-PA nor glucose was fully depleted. The remaining concentration of glucose was 27 ± 1.6 mM and the oxygen concentration of 80% can be ruled out as limiting factor for further growth. The conversion rates of β-PA and the growth rate µ and volumetric growth rate rx were compared in Table 1. The µ_max_ was determined for PsJN after 17.9 h of cultivation with 0.14 h^−1^, which is equate to a doubling time (t_d_) of 4.95 h. In contrast, the strain BS115 reached a µ_max_ of 0.23 h^−1^ after 27.0 h of cultivation.

**Table 1:** Characterization of *Paraburkholderia* sp. fermentation processes

	Max. growth rate µ_max_ (1/h)	Max. volumetric growth rate rx [g/(L h)]	Max. (*S*)-β-PA volumetric consumption rate Q_(*s*)-PA_ (mM/h)	Max. specific (*S*)-β-PA consumption rate q_(*S*)-PA_ [mmol/(g h)]	t_d_ (h)
*Paraburkholderia phytofirmans* PsJN	0.14	0.036	0.21	− 2.1	4.95
*Paraburkholderia* sp. BS115	0.23	0.063	0.26	− 1.3	3.0

The calculations are based on the mean values of three independent fermentations

**Fig. 2:**
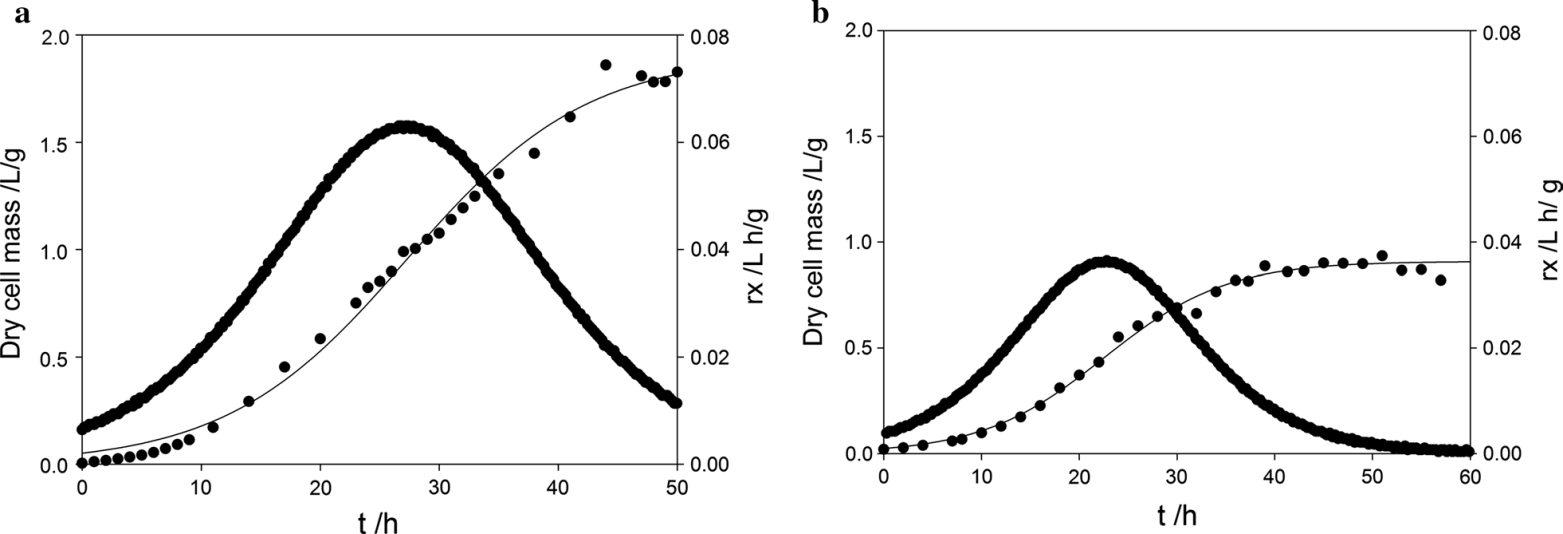
Dry cell mass building rate of **a** BS115 and **b** PsJN. The dry cell mass data points were fitted with a three parameter sigmoid function with an R^2^ of 0.99. This function was used to calculate the slope (ΔDCM/Δt)

**Fig. 3:**
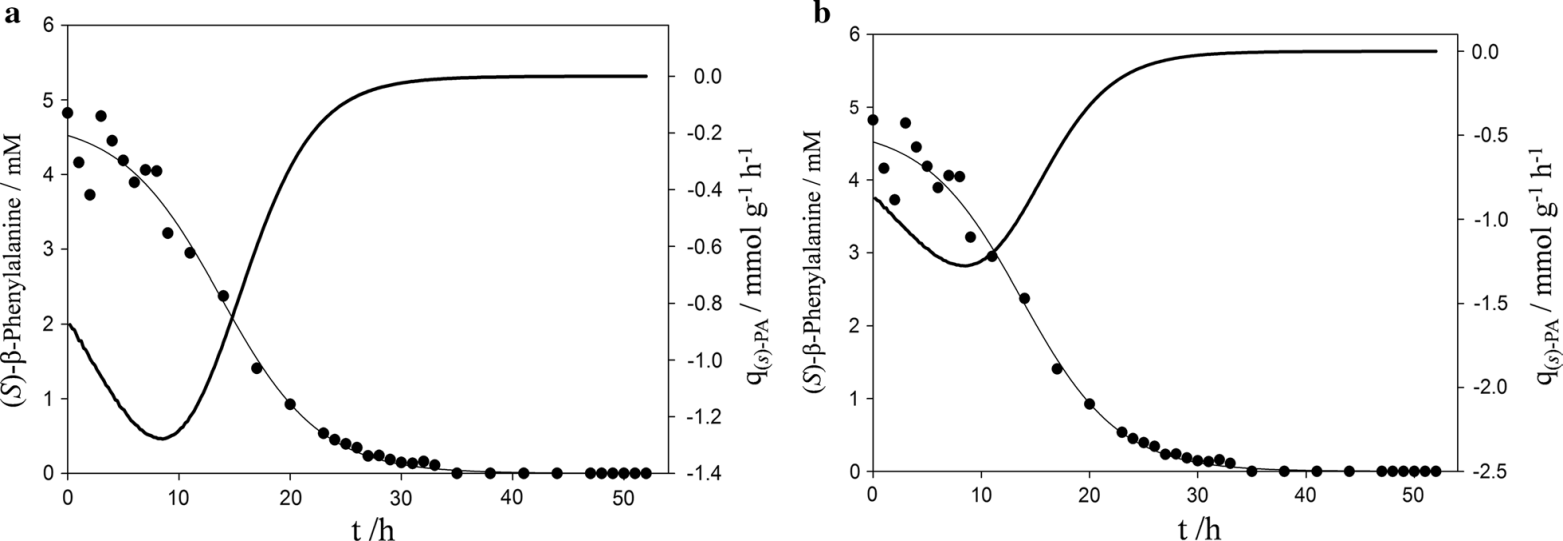
Consumption rate of (*S)*-β-PA. Bold line—q_(*s*)-PA_. Points with line-concentration of (*S)*-β-PA in bioreactor. **a** BS115, **b** PsJN

During fermentation BS115 grew 1.6 times faster than PsJN on β-PA (Fig. 2). The Q_(*s*)-PA_ rate was calculated to be 0.26 mM/h for BS115 after 14.2 h (Fig. 3). The Q_(*s*)-PA_ rate of PsJN was slightly lower with 0.21 mM/h after 16.2 h of cultivation time. In addition the q_(*s*)-PA_ was determined for both strains, which is correlated to the biomass concentration. On the other hand BS115 had a specific q_(*s*)-PA_ rate which is slightly lower with 0.74 mmol/g/h compared to PsJN with 0.88 mmol/g/h (Fig. 3).

PsJN produced less biomass but the cells converted (*S*)-β-PA more efficiently. PsJN started to convert with maximal specific consumption rate, whereas BS115 reached its maximal consumption rate after 8 h of cultivation time. In contrast to (*S*)-β-PA, the consumption rate of (*R*)-β-PA could not be fitted for BS115, caused by the high variances and inconstant decreasing rate between 25 and 50 h of cultivation. The volumetric production rate Q_AP_ of AP reached a maximum of 0.07 mM/h for PsJN after 10.8 h of fermentation. After several hours the AP concentration decreased with a much slower with a Q_AP_ rate of 0.01 mM/h. Initially, the BS115 Q_AP_ rate was faster than calculated for PsJN, with 0.34 mM/h.

### ω-Transaminase activity test

The β-PA degrading activity of both strains were tested with cell free lysate from BS115 and compared to PsJN lysate. The temperature was set to 30 °C according to the cultivation temperature of BS115 and PsJN. For the transamination reaction 0.1 mM of the ω-TA cofactor molecule PLP and selected α-ketoglutarate as acceptor molecule was added due to the compatibility towards well characterized β-phenylalanine transaminases (Wybenga et al. 2012; Crismaru et al. 2013). The final concentration of applied protein in the reaction mixture was 0.47 mg/mL for PsJN and 0.95 mg/mL for BS115. The concentration of *rac*-β-PA in the lysate-reaction mixture was determined at different times, to recognize slow and fast conversions as well as long-term effects (Additional file 1: Figure S3). After 24 h both lysates showed only activity for (*S*)-β-PA and a complete conversion of the (*S*)-enantiomer. The transaminase activity at the beginning of the reaction was considerably higher for PsJN lysate than for BS115. The specific activities of both preparations were calculated between samples of 15 s and 1 h and varied between 35 ± 5 mU/mg for PsJN and 8 ± 4 mU/mg for BS115. In total, the amounts of the obtained lysate activity was 1.25 U and 0.32 U in 100 mL cell culture at an OD_600_ of 3.1.

### Characterization of BS115 ω-TA

The ω-TA activity of BS115 was characterized. The activity of the putative enzyme was higher in a slightly alkaline TRIS-buffer system at pH 8. In addition to α-ketoglutarate the BS115 lysate also showed activity towards the amino acceptors pyruvate and oxaloacetate. However, for the latter acceptor molecules the activity was 50% lower compared to α-ketoglutarate. Moreover, derivatives of β-PA were tested as amino donors. The transaminase activity was higher towards non-substituted β-PA and in contrast relatively low for β-PA with a chlorine in *para* position at the phenyl-ring. Also remarkable is that the enantioselectivity decreased only for the strong polar nitro-substituent (Table 2).

**Table 2:** Substrate spectrum of putative BS115 ω-TA

Amino-donor	Relative activity (%)	ee
*rac*-3-amino-3-phenylpropionic acid (β-PA)	100	> 99%
*rac*-3-amino-4-phenylbutyric acid (β-homophenylalanine)	0	–
*rac*-3-amino-3-(4-methoxyphenyl)propionic acid	46	> 96%
(*S*)-3-amino-3-(4-hydroxyphenyl)propionic acid (β-tyrosine)	69	–
*rac*-3-amino-3-(4-fluorophenyl)propionic acid	61	> 99%
*rac*-3-amino-3-(4-nitrophenyl)propionic acid	34	> 91%
*rac*-3-amino-3-(4-chlorophenyl)propionic acid	19	> 99%

The reactions were carried out in 50 mM TRIS-buffer at pH 8, with an acceptor molecule concentration of 5 mM. The total protein-concentration of the cell lysate was set to 0.2 mg/mL. ee% was determined after 30 min of reaction time

By contrast, the putative ω-TA from BS115 showed no activity towards *rac*-β-homophenylalanine, which comprises only one additional carbon atom between the amino group and phenyl ring. In the case of variation of the substituents of the phenyl-ring of β-PA, the enzymes showed a wide promiscuity.

## Discussion

### Degradation of (*S*)-β-PA by BS115 and PsJN

Both *Paraburkholderia* strains BS115 and PsJN were able to grow with (*S*)-β-PA as sole source of nitrogen. The metabolization of the N-source most likely occurs via a transaminase reaction in which the amino group of (*S*)-β-PA is transferred to an α-keto acid. Although α-ketoglutarate has been shown to be the most suitable in vitro amino acceptor, the in vivo application of other molecules like pyruvate is also possible.

Deracemization of β-PA by living cells has only once been reported so far: The only comparable fermentation example from Mano et al. showed that the soil bacterium *Variovorax* sp. JH2 is able to convert (*S*)-selective 61 mM of β-PA within 8 days (Mano et al. 2006). They achieved a maximal degradation rate of 0.26 mM/h towards (*S*)-β-PA, which is comparable to the uptake rates of PsJN and BS115. The approach to utilize living cells for chiral resolution of *rac*-β-PA should be tested in further experiments at larger scales and at higher concentration levels, to prove whether a microbial process might be able to compete with established industrial deracemization processes using lipases demonstrated by former Evonik-Degussa company (Grayson et al. 2011). Yeast cells have successfully been used for the chiral resolution of alcohols and more recent examples document the microbial resolution e.g. of dl-glyceric acid (Glänzer et al. 1987; Kometani et al. 1995; Sato et al. 2015).

**Fig. 4:**
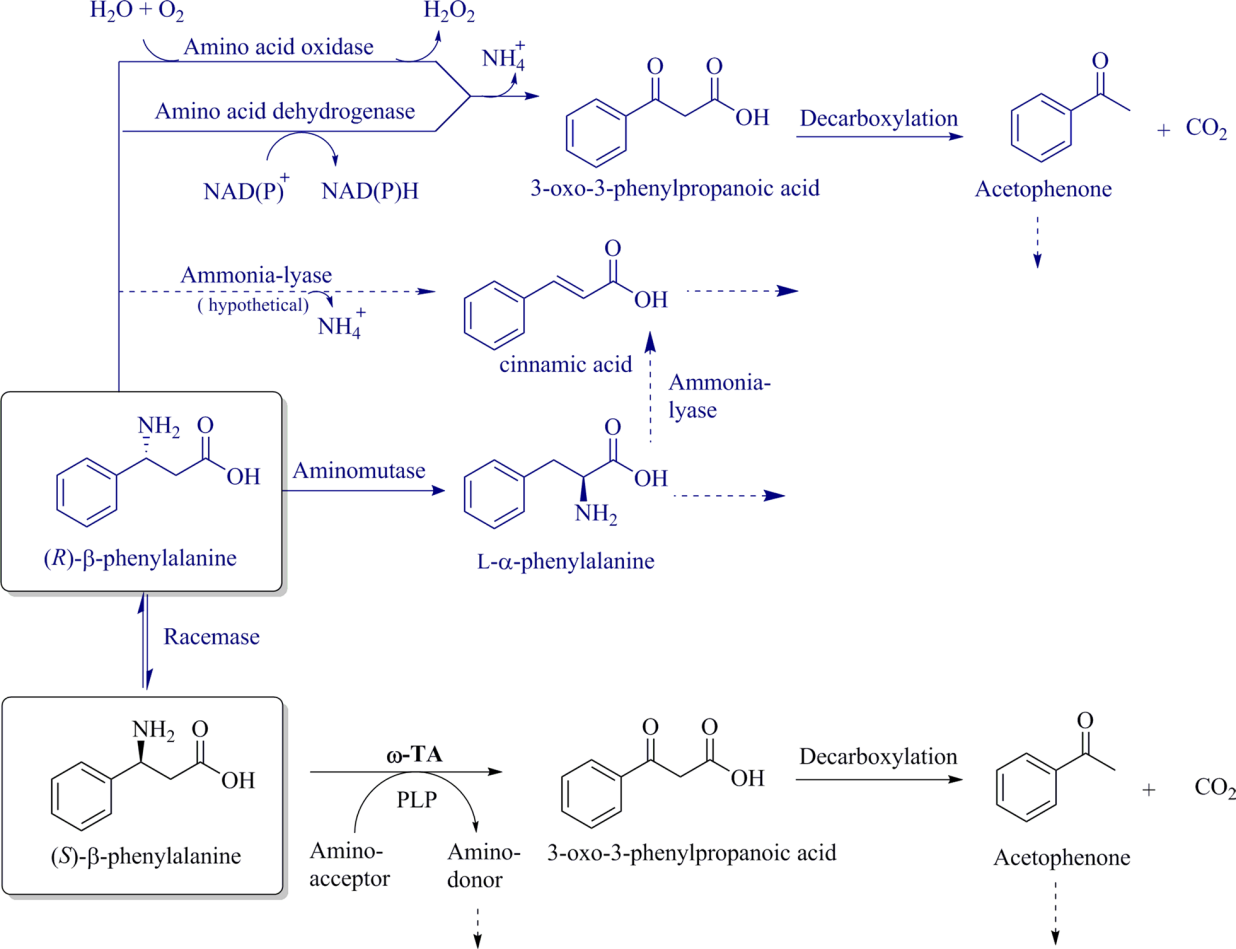
Putative degradation mechanisms of (*R*)-β-PA. In blue: putative degradation mechanisms of (*R*)-β-PA. Since AP no longer emerges when (*R*)-β-PA is consumed but rather decreases during fermentation (see again Additional file 1: Figure S1a), the reactions via aminomutase appear to be more likely. Bacterial ammonia lyases (dashed arrow) with activity towards β-PA have not yet been reported. In black: identified degradation pathways of (*S*)-β-PA. The degradation of (*S*)-β-PA occurs via transamination and leads to the formation of AP reciprocal to (*S*)-β-PA decline (as shown in Additional file 1: Figure S1a)

The rapid degradation of the (*S*)-enantiomer, in contrast to the (*R*)-enantiomer, resulted in a reduction of the pH-value by metabolizing almost half of the d-glucose. According to this, the final pH value of 3.5 is most likely to be limiting. In addition it could be seen that during BS115 fermentation the pH-value stagnated at a constant value of 5 for several hours before it dropped to 3.5. A total consumption of the (*R*)-enantiomer might be possible, if the fermentation process was pH regulated. The fermentation parameters quantified are valuable for using the strains as microbial target to investigate metabolic pathways of the *rac*-β-PA metabolization to ultimately achieve the microbial synthesis of β-PA, as has already been established for α-amino acids (Sanchez et al. 2017). In contrast the experiments lead to the assumption, that BS115 is able to convert (*R*)-β-PA by a so far unknown mechanism (Fig. 4). The lowering of the pH value indicates that either an organic acid is formed or a previously buffering substance [e.g. β-PA (pI 5.84)] has been consumed by BS115. β-PA could thus itself act as a buffer at pH 5.8, but continuing degradation leads to further acidification due to the resulting β-keto acid production.

### By-product analysis

The proposed transamination of (*S*)-β-PA leads to the corresponding β-keto acid 3-oxo-3-phenylpropanoic acid. However, this molecule has never been detected in medium as it spontaneously decarboxylates to AP as has been shown in previous studies (Crismaru et al. 2013; Dold et al. 2016). AP on the other hand was shown to emerge in parallel to the degradation of (*S*)-β-PA, peaking for both strains after about 30 h when (*S*)-β-PA is depleted (Additional file 1: Figure S1). Progress of AP formation rate and (*S*)-β-PA degradation rate are nearly identical for both strains which is strong evidence for the proposed transamination mechanism (Fig. 4). In BS115, even the molar concentrations of emerging AP and metabolized (*S*)-β-PA are close to identical. So it is most likely that after transamination the corresponding β-keto acid decarboxylates and the emerging AP is excreted.

After depletion of (*S*)-β-PA, AP concentration in the medium notably decreases. Substantial evaporation of the volatile AP can be excluded from control experiments (data not shown). A reuptake of AP is possible, but from the data at hand it cannot be stated whether AP serves as additional carbon source or as amino acceptor without further metabolization which would also lead to the observed depletion. Rehdorf et al. showed in *Pseudomonas putida* strain JD1, that a Baeyer–Villiger monooxygenase converts 4-hydroxy AP to phenyl-acetate. Finally, phenyl-acetate is further degraded via a β-ketoadipate metabolic pathway. At the same time, the enzyme is also able to convert AP (Rehdorf et al. 2009). This metabolic pathway releases acetic acid, which should lower the pH value of the fermentation medium. This is supported by the fact that at the very time when acetophenone decreases drastically (after 40 h), also the pH value of BS115 fermentation decreases from 6 to 4 (Additional file 1: Figure S4).

Further experiments with a different *Paraburkholderia* strain demonstrated that AP can be reduced by a carbonyl reductase (Singh et al. 2009), which might serve as an evidence for further metabolization inside the cells. It is also known that *Burkholderia* sp. expresses a reductase which is able to reduce 2-aminoacetophenone to 2-amino-1-phenylethanol (Yamada-Onodera et al. 2007). The same reductase might also be able to convert AP. Furthermore PsJN is known to degrade even more complex aromatic biomolecules like the plant hormone indole-3-acetic acid by a rather complex degradation pathway to catechol (Donoso et al. 2017). So a bacterial metabolization of AP as a reason for its decrease is most likely.

### Superior growth of BS115

Although both strains clearly metabolize (*S*)-β-PA via the same mechanism, BS115 reaches a final OD_600_ twice as high as that of PsJN under identical growth conditions. According to 16S-rDNA sequencing, both strains show > 99% identity, so the basic metabolism might not differ too much and maybe only one additional enzyme makes up the severe difference in growth. However, it is also possible that different enzymes from other microbial sources are integrated in BS115 genome via horizontal gene transfer, especially when the strain was exposed to extraordinary amino sources in soil. Since the PsJN cultures obviously reach the stationary phase when (*S*)-β-PA is depleted although sufficient amounts of d-glucose are still at hand, a growth stop can be explained by the inability of this strain to use the (*R*)-enantiomer as nitrogen source; (*R*)-β-PA remains completely unconsumed during the whole fermentation. A growth inhibition by other factors like toxic metabolites is unlikely since the closely related strain BS115 very probably faces similar but even stronger stress conditions (e.g. ongoing acidification, depletion of other nutrients than N-source).

Moreover, BS115 shows a quite similar cell dry mass per depleted (*S*)-β-PA minus depleted (*R*)-β-PA. In total BS115 built 1.82 g of biomass per liter and consumed 7.72 mM of *rac*-β-PA, that is a ratio of 4.3 g/(mM(β-PA)). In contrast PsJN built only 0.82 g/L (ratio 5.9 g/(mM(β-PA)) and was at the same time more efficient in using only (*S*)-PA. So we postulate at least one additional enzyme in BS115 which allows the metabolization or (*R*)-β-PA as additional N-source and potentially also as additional C-source, leading to a better growth and to the formation of considerably more cell dry mass from the same medium.

### Degradation of (*R*)-β-PA by BS115

Several options of amino acid metabolization are identified and well investigated (see also “Introduction” section), and most of these also seem conceivable for the degradation of (*R*)-β-PA. Since activity has only been observed in growing cells, a cofactor-dependent mechanism is likely. Moreover, an additional transaminase would have been detected under the given conditions, and also a side activity of the identified (*S*)-β-PA can be excluded: (*R*) and (*S*)-ω-TAs show only low sequence identities and can clearly be differentiated by protein fold types (Höhne et al. 2010; Buß et al. 2018a). A transamination of (*R*)-β-PA would have led to the formation of AP, but on the contrary the concentration of AP decreased. Several (*R*)-selective ω-TAs have been described for the ability to convert and synthesize (*R*)-configurated amines. However, activity towards aromatic or bulky β-AAs were only observed for (*S*)-selective ω-TAs (Koszelewski et al. 2010; Seo et al. 2012; Dold et al. 2016) and no (*R*)-PA converting transaminase has been reported until now (Mutti et al. 2011; Mallin et al. 2014; Jiang et al. 2015; Skalden et al. 2015). Only Mano et al. investigated an (*R*)-selective strain, *Arthrobacter* sp. AKU 638, which is suitable for chiral resolution to gain the optical pure (*S*)-β-PA. However, this degradation process is very slow and took 13 days of fermentation and in contrast to BS115 they did not recognize any depletion of the (*S*)-enantiomer at the same time (Mano et al. 2006). Oxidative deamination has been suggested, but until now, the mechanism for the degradation of (*R*)-β-PA in *Arthrobacter* sp. AKU 638 is uncovered. Only recently it was also shown that the ethyl ester of (*R*)-PA can be converted by a commercial fold type IV transaminase (ATA117 11Rd), which however has been largely modified and no longer shows a high identity to wild-type ω-TA (Buß et al. 2018b). Therefore, it seems unlikely that such a transaminase can occur in BS115, especially since tests with cell lysate do not indicate any transaminase reaction.

In fact, the metabolism of (*R*)-β-PA has only been reported for microorganism with assured aminomutases or ammonia lyases genes (Szymanski et al. 2009; Jiang et al. 2015; Weise et al. 2015). The activity of aminomutases is described as quite low which might explain the rather slow degradation of (*R*)-β-PA using BS115 (Bartsch et al. 2013). Also several amino acid oxidases exist with high activity towards several amines (Alexeeva et al. 2002; Ghislieri et al. 2013), but as yet amino acid oxidases with activity towards β-PA have not been published. Analogously no wildtype β-amino acid dehydrogenase is known; the only example of a dehydrogenase with activity towards β-PA is an engineered enzyme from the amino acid fermenting bacterium *Candidatus cloacamonas* (Zhang et al. 2015).

However, PA-racemases have been described for the racemization between l and d-α-PA in bacteria (Conti et al. 1997) and for plants the *Taxus* (yew) PA aminomutase is known to use α-phenylalanine as substrate to produce (*R*)-β-PA (Cox et al. 2009). In contrast, no bacterial β-phenylalanine lyase was reported so far, so it is rather unlikely that β-PA is degraded by a lyase; however, this possibility is shown in Fig. 4 for completeness. Wu et al. showed that a PA mutase from *Taxus chinensis* can be utilized for chiral separation of *rac*-β-PA. The corresponding α-PA is then degraded to cinnamic acid using an α-PA ammonia lyase from *Rhodosporidium toruloides*. The reported reaction time for chiral resolution was quite long with more than 48 h using 2 mM of substrate (Wu et al. 2010). Apart from this also β-tyrosine aminomutase is known from *Oryzae sativa* with high enantioselectivity towards (*R*)-β-PA (Walter et al. 2016). So a slow enzymatic racemization of (*R*)-PA to (*S*)-PA and further metabolization of the latter by BS115 appears at least possible; but no bacterial aminomutase with such an activity is known until now.

### Hypothetical degradation pathway for (*R*)-β-PA

Recently Csuka et al. (2017) reported that *Pseudomonas fluorescens* R124 encodes three different class I lyase like enzymes, namely an ammonia-lyase, an aromatic 2,3 aminomutase and a histidine ammonia-lyase. The authors describe, that under nitrogen limitation, *P. fluorescens* is able to integrate new genes by horizontal gene transfer to overcome limitations. Therefore BS115 might be adapted towards special nitrogen limitations, even when the genome is quite similar to PsJN. For this reason it might be possible that BS115, in the presence of (*S*)-β-PA, expresses an ammonia-lyase or 2,3 aminomutase which could convert (*R*)-β-PA to cinnamic acid. The transmutation of an ammonia-lyase into a 2,3 aminomutase is possible by a single mutation and can change the functionality of a mutase to a lyase (Bartsch et al. 2013). The vice versa functionality change of a lyase to a mutase has not yet been reported, but attempts have been made to determine the decisive amino acid residues using mutation studies of e.g. Attanayake et al. (2018). Bacterial aminomutase with β-PA activity would also be rather unexpected, although not unthinkable, considering that horizontal gene shifts are possible. In this case, any cinnamic acid produced could be largely metabolized. This pathway has been shown in *Pseudomonas* strains where cinnamic acid is metabolized to *o*-hydroxyphenylpropionic acid and to 2,3-dihydroxyphenylpropionic acid (Blakley and Simpson 1964). BS115 might be able to regenerate NAD(P)^+^ by this way using an NADP oxidoreductase. In high concentrations cinnamic can also be reduced by NADH using fumarate reductase or by an oxygen-sensitive 2-enoate reductase (Hillier et al. 1979; Sun et al. 2016). An oxygen-sensitive enzyme would also give an explanation why the activity is hard to detect in crude extracts without further protective measures.

**Fig. 5:**
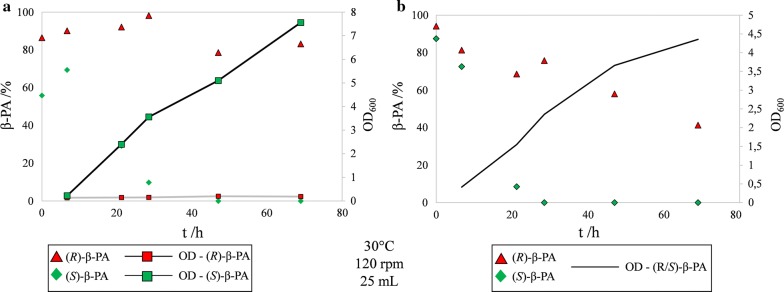
Conversion of (*R*)-β-PA in presence of the (*S*)-enantiomer (**b**) and no conversion as sole nitrogen source (**a**). The experiment was performed using optically pure β-PA in minimal medium. BS115 was pre-cultivated on (*R*/*S*)-β-PA for 3 days in shaking-flaks

This assumption is supported by growth experiments on enantiopure (*R*) and (*S*)-β-PA as nitrogen sources, as BS115 is only able to degrade (*R*)-β-PA in the presence of (*S*)-β-PA and not when added as sole N-source (Fig. 5). The experiments showed that (*S*)-β-PA was completely converted, whereas the (*R*)-enantiomer as sole nitrogen source remained untouched even after a cultivation duration of 70 h (Fig. 5a). On the other hand, the (*R*)-enantiomer was substantially degraded in the presence of (*S*)-β-PA (Fig. 5b). Only 40% of 10 mM (*R*)-enantiomer retained within the cultivation medium after 70 h. Since growth continues after complete depletion of (*S*)-β-PA, (*R*)-β-PA is obviously made accessible as nitrogen source by one of the mechanisms depicted in Additional file 1: Figure S4. Moreover (*R*)-β-PA might also serve as additional carbon source or as electron acceptor if cinnamic acid is produced.

All of the above discussed potential pathways and metabolization steps using the addressed different enzymes are also summarized in Fig. 4. These results also confirm the report from Mano et al., that no activity can be detected in cell-free lysate of an *Arthrobacter* sp. AKU 638 strain for (*R*)-β-PA consumption. Furthermore, the reported very slow degradation, might hint to a similar mechanism using the same enzyme as in BS115.

We characterized the degradation of racemic β-PA by a 3 days fermentation of two *Paraburkholderia* strains PsJN and BS115 in a 2.5 L-benchtop fermenter in 1 L medium. PsJN exhibited strict (*S*)-selectivity and therefore can be utilized as whole cell biocatalyst to obtain (*R*)-β-PA in high optical purity by chiral resolution. The spontaneous decarboxylation of the emerging β-keto acid to acetophenone, for the first time documented over the whole degradation process, shifts the equilibrium irreversibly towards the desired direction. However, as a process with industrial relevance a high cell density fermentation has to be developed with medium conditions allowing much higher substrate concentrations, which is currently limited by the low water solubility of β-PA. Therefore an alternative would be to perform a fed batch fermentation. After uncovering the degradation pathway of (*S*)- and (*R*)-β-PA in *Paraburkholderia* sp., these strains might be optimized for the production of β-PA as has already been shown for several α-amino acid production strains (Sanchez et al. 2017). The abilities to synthesize (*R*)-configurated aromatic amines and amino acids by asymmetric synthesis are limited by the availability of (*R*)-selective enzymes; so far, only few plant enzymes are known for the synthesis of (*R*)-β-PA (Ratnayake et al. 2016; Buß et al. 2018b). So the investigation of the (*R*)-β-PA degradation process in BS115 might not only lead to a deeper understanding of the biochemical pathways of β-AA but to novel approaches for the chemoenzymatic synthesis of this highly relevant substance class.

## Additional file

**Additional file 1: Figure S1.** Acetophenone (AP) content during fermentation process in comparison to (*S*)-β-PA concentration. AP concentration rises inversely proportional to (*S*)-β-PA degradation and decreases after *(S)*-β-PA depletion. a) BS115 fermentation b) PsJN fermentation. **Figure S2.** Extracellular capsule built by BS115 during fermentation after depletion of (*S*)-β-PA. The capsule of BS115 was visualized by negative contrasting with Chinese ink. **Figure S3.** Transaminase activity of cell free lysate of BS115 and PsJN. In red triangles: (*R*)-β-PA; in green triangles (*S*)-β-PA. The reaction was performed at 30 °C in reaction mixture (see also section 2.6) of 12 mM of *rac-* β-PA using α-ketoglutarate as amino acceptor. The reaction and sampling time was chosen to depict fast reactions as well as possible long-term effects. **Figure S4.** pH profile during fermentation of BS115 and PsJN in 1.5 L bioreactor systems using minimal medium. In contrast to PsJN, BS115 showed a stabilization of the pH value between 20 and 40 h. **Figure S5.** Chiral separation of β-PA using IBLC-OPA pre-column derivatization and reversed phase HPLC according to Brucher et al. (2010a).The retention time of the (*R*)-enantiomer is 3.7 min, of the (*S*)-enantiomer 4.8 min. **Figure S6.** Relative transaminase activity of BS115 in regard to pH. The reactions were performed with 2.5 mM of racemic β-phenylalanine using 0.5 mg/mL of protein at 30 °C.

## Abbreviations

β-AA: β-amino acid
β-PA: β-phenylalanine
AP: acetophenone
Q-AP: volumetric production rate AP
Q_(s)-PA_: volumetric consumption rate (*S*)-β-PA
q_(s)-PA_: specific consumption rate
µ_max_: max. growth rate
t_d_: doubling time
Y_x/s_: biomass yield per substrate amount
r_x_: volumetric growth rate
