# Supplementary material for: Enantiomer discrimination in β-phenylalanine degradation by a newly isolated Paraburkholderia strain BS115 and type strain PsJN
Source: AMB Express. 2018 Sep 21;8:149. doi: 10.1186/s13568-018-0676-2 (PMC6150868; doi:10.1186/s13568-018-0676-2)
Supplement: Supplementary file 1 — Additional file 1: Figure S1. Acetophenone (AP) content during fermentation process in comparison to (S)-β-PA concentration. AP concentration rises inversely proportional to (S)-β-PA degradation and decreases after (S)-β-PA depletion. a) BS115 fermentation b) PsJN fermentation. Figure S2. Extracellular capsule built by BS115 during fermentation after depletion of (S)-β-PA. The capsule of BS115 was visualized by negative contrasting with Chinese ink. Figure S3. Transaminase activity of cell free lysate of BS115 and PsJN. In red triangles: (R)-β-PA; in green triangles (S)-β-PA. The reaction was performed at 30 °C in reaction mixture (see also section 2.6) of 12 mM of rac- β-PA using α-ketoglutarate as amino acceptor. The reaction and sampling time was chosen to depict fast reactions as well as possible long-term effects. Figure S4. pH profile during fermentation of BS115 and PsJN in 1.5 L bioreactor systems using minimal medium. In contrast to PsJN, BS115 showed a stabilization of the pH value between 20 and 40 h. Figure S5. Chiral separation of β-PA using IBLC-OPA pre-column derivatization and reversed phase HPLC according to Brucher et al. (2010a).The retention time of the (R)-enantiomer is 3.7 min, of the (S)-enantiomer 4.8 min. Figure S6. Relative transaminase activity of BS115 in regard to pH. The reactions were performed with 2.5 mM of racemic β-phenylalanine using 0.5 mg/mL of protein at 30 °C. [file 13568_2018_676_MOESM1_ESM.pdf]

Supplementary Material

Enantiomer discrimination in β-phenylalanine degradation by a newly isolated Paraburkholderia strain BS115 and type strain PsJN

Oliver Buß*, Sarah-Marie Dold, Pascal Obermeier, Dennis Litty, Delphine Muller, Jens Grüninger and Jens Rudat

*** Correspondence:** Corresponding Authors: oliver.buss@kit.edu, jens.rudat@kit.edu

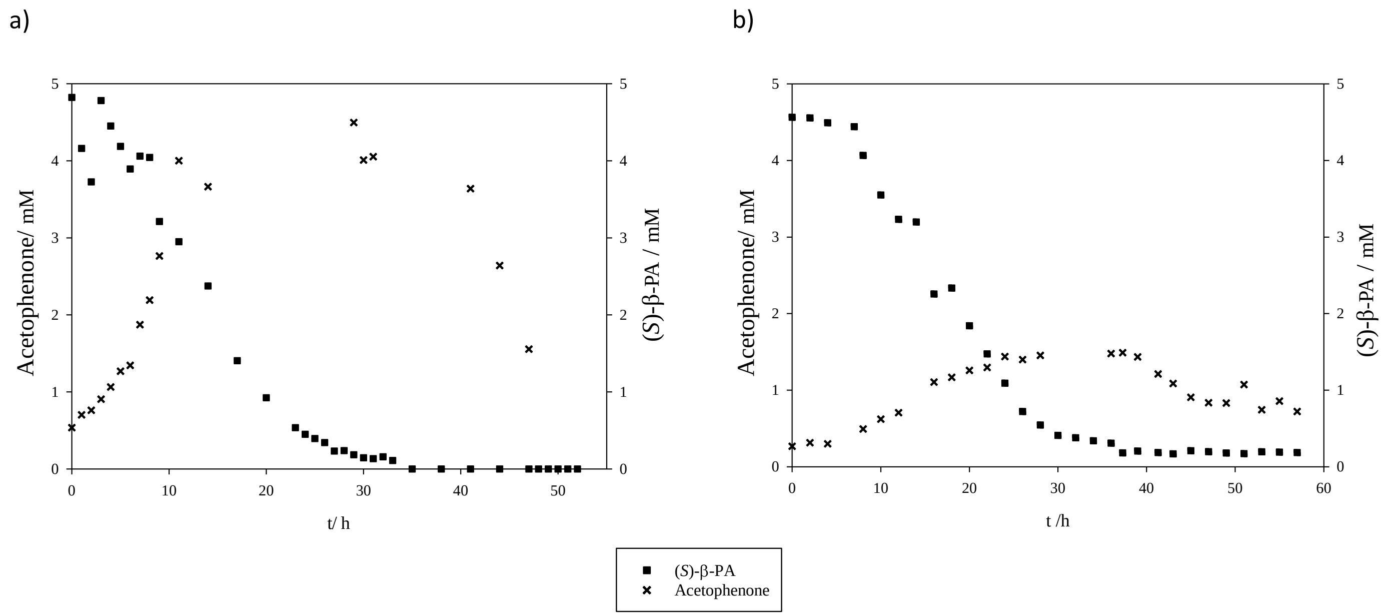

Figure S1. Acetophenone (AP) content during fermentation process in comparison to (*S*)-β-PA concentration. AP concentration rises inversely proportional to (*S*)-β-PA degradation and decreases after *(S)*-β-PA depletion. a) BS115 fermentation b) PsJN fermentation.

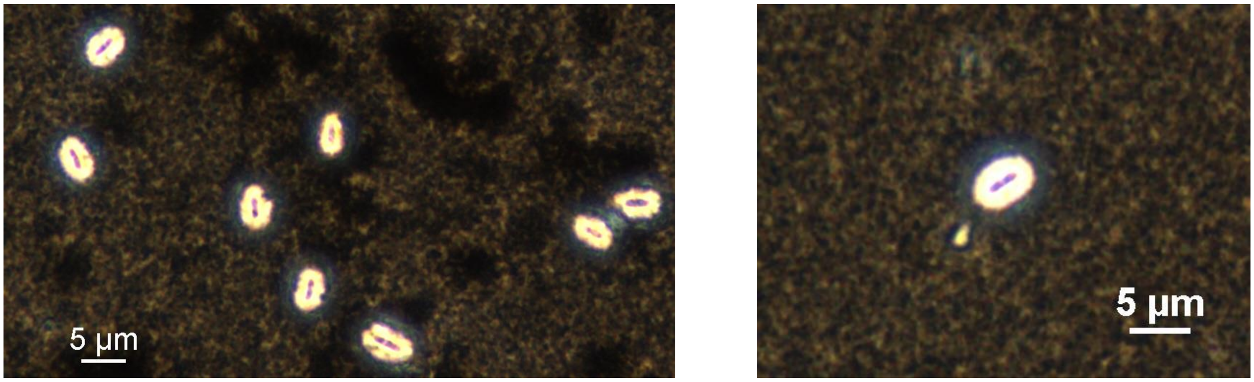

Figure S2. Extracellular capsule built by BS115 during fermentation after depletion of (*S*)-β-PA. The capsule of BS115 was visualized by negative contrasting with Chinese ink.

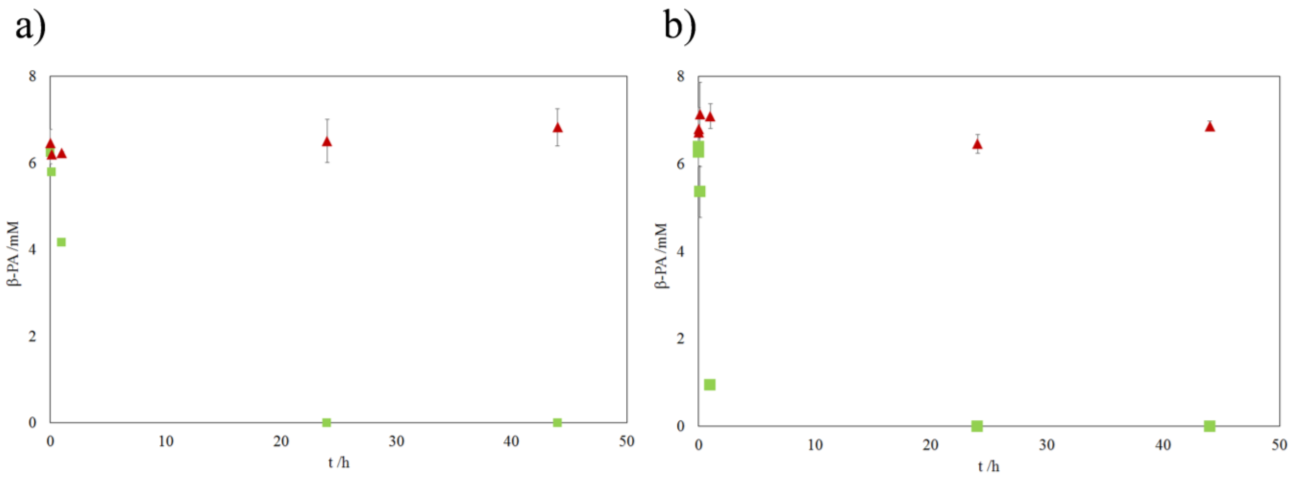

Figure S3. Transaminase activity of cell free lysate of BS115 and PsJN. In red triangles: (*R*)-β-PA; in green triangles (*S*)-β-PA. The reaction was performed at 30°C in reaction mixture (see also section 2.6) of 12 mM of *rac*- β-PA using α-ketoglutarate as amino acceptor. The reaction and sampling time was chosen to depict fast reactions as well as possible long-term effects.

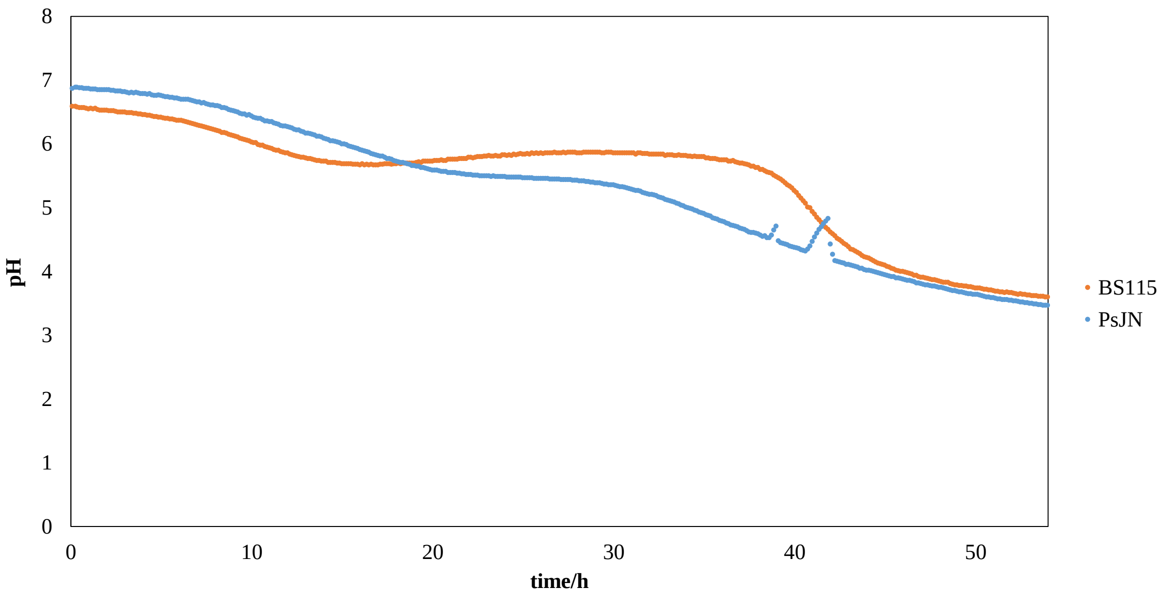

Figure S4. pH profile during fermentation of BS115 and PsJN in 1.5 L bioreactor systems using minimal medium. In contrast to PsJN, BS115 showed a stabilization of the pH value between 20 and 40 h.

(*R*)-β-Phenylalanine 5 mM

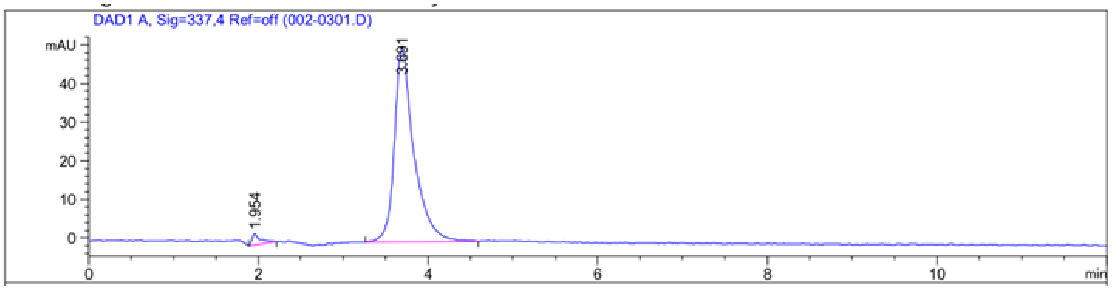

(*S*)-β-Phenylalanine 5 mM

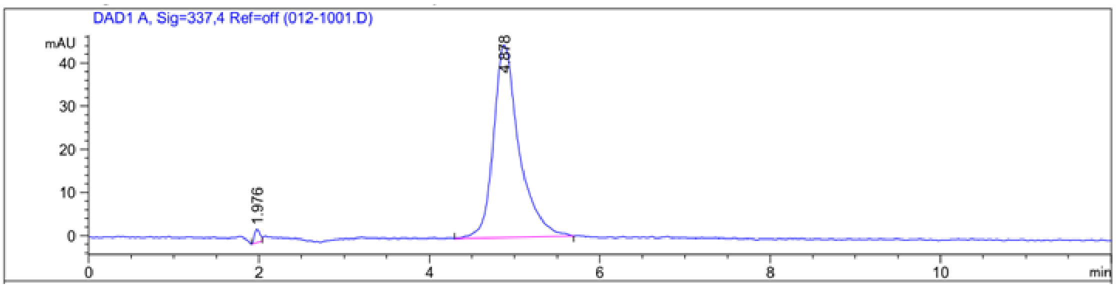

Racemate 10 mM

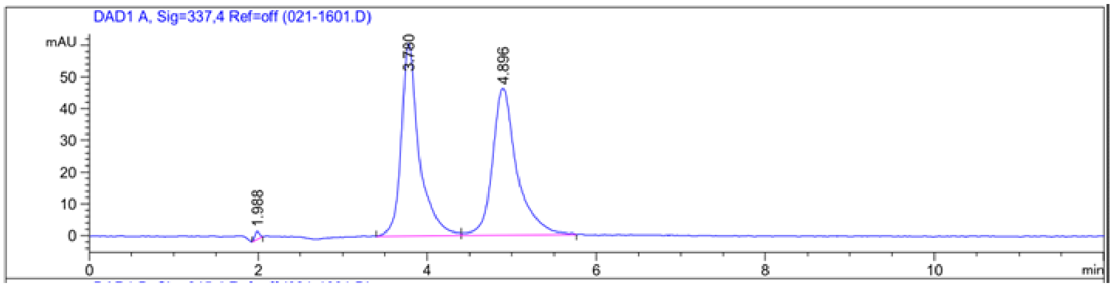

Fermentation sample

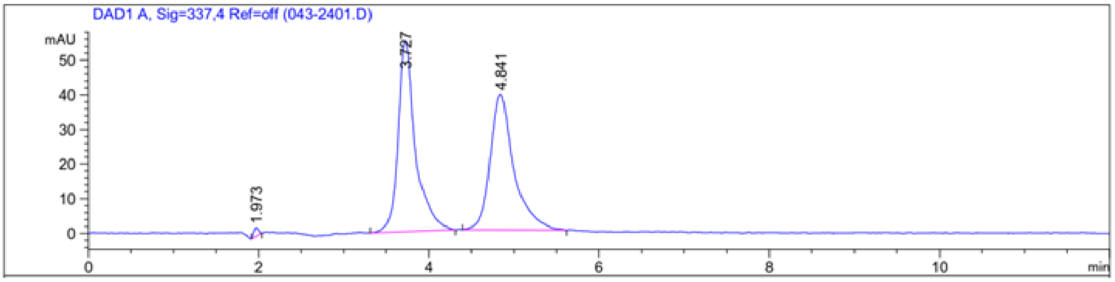

Figure S5. Chiral separation of β-PA using IBLC-OPA pre-column derivatization and reversed phase HPLC according to Brucher et al. (2010a).The retention time of the (*R*)-enantiomer is 3.7 min, of the (*S*)-enantiomer 4.8 min.

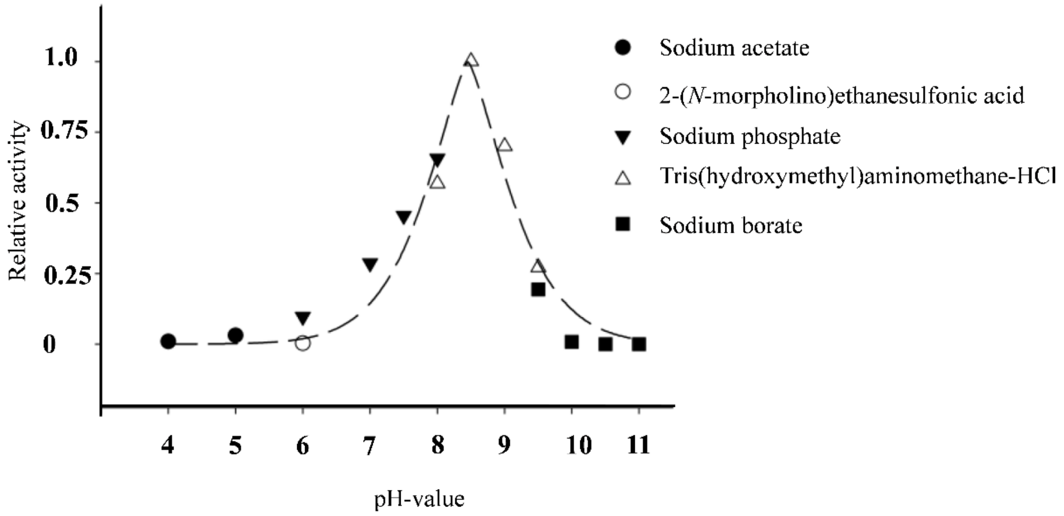

Figure S6. Relative transaminase activity of BS115 in regard to pH. The reactions were performed with 2.5 mM of racemic β-phenylalanine using 0.5 mg/mL of protein at 30°C. (Figure was adapted from doctoral thesis of (Brucher, 2011).

References

Brucher, B. (2011). Novel Transaminases for the Biocatalytic Synthesis of Aromatic β-Amino Acids. Karlsruhe Institute of Technology.

Brucher, B., Rudat, J., Syldatk, C., and Vielhauer, O. (2010). Enantioseparation of Aromatic β^3-Amino acid by Precolumn Derivatization with *o*-Phthaldialdehyde and *N*-Isobutyryl-L-cysteine. *Chromatographia* 71, 1063–1067. doi:10.1365/s10337-010-1578-x.
